# Simple rapid in vitro screening method for SARS-CoV-2 anti-virals that identifies potential cytomorbidity-associated false positives

**DOI:** 10.1186/s12985-021-01587-z

**Published:** 2021-06-09

**Authors:** Kexin Yan, Daniel J. Rawle, Thuy T. Le, Andreas Suhrbier

**Affiliations:** 1 QIMR Berghofer Medical Research Institute, Brisbane, QLD 4029 Australia; 2 Australian Infectious Disease Research Centre, GVN Center of Excellence, Brisbane, QLD 4029 and 4072 Australia

**Keywords:** SARS-CoV-2, Drug screening, Cytopathic effect, Cytotoxicity, Cytomorbidity

## Abstract

**Background:**

The international SARS-CoV-2 pandemic has resulted in an urgent need to identify new anti-viral drugs for treatment of COVID-19. The initial step to identifying potential candidates usually involves in vitro screening that includes standard cytotoxicity controls. Under-appreciated is that viable, but stressed or otherwise compromised cells, can also have a reduced capacity to replicate virus. A refinement proposed herein for in vitro drug screening thus includes a simple growth assay to identify drug concentrations that cause cellular stress or “cytomorbidity”, as distinct from cytotoxicity or loss of viability.

**Methods:**

A simple rapid bioassay is presented for antiviral drug screening using Vero E6 cells and inhibition of SARS-CoV-2 induced cytopathic effects (CPE) measured using crystal violet staining. We use high cell density for cytotoxicity assays, and low cell density for cytomorbidity assays.

**Results:**

The assay clearly illustrated the anti-viral activity of remdesivir, a drug known to inhibit SARS-CoV-2 replication. In contrast, nitazoxanide, oleuropein, cyclosporine A and ribavirin all showed no ability to inhibit SARS-CoV-2 CPE. Hydroxychloroquine, cyclohexamide, didemnin B, γ-mangostin and linoleic acid were all able to inhibit viral CPE at concentrations that did not induce cytotoxicity. However, these drugs inhibited CPE at concentrations that induced cytomorbidity, indicating non-specific anti-viral activity.

**Conclusions:**

We describe the methodology for a simple in vitro drug screening assay that identifies potential anti-viral drugs via their ability to inhibit SARS-CoV-2-induced CPE. The additional growth assay illustrated how several drugs display anti-viral activity at concentrations that induce cytomorbidity. For instance, hydroxychloroquine showed anti-viral activity at concentrations that slow cell growth, arguing that its purported in vitro anti-viral activity arises from non-specific impairment of cellular activities. The cytomorbidity assay can therefore rapidly exclude potential false positives.

**Supplementary Information:**

The online version contains supplementary material available at 10.1186/s12985-021-01587-z.

## Main text

The global SARS-CoV-2 pandemic has resulted in widespread activities seeking to identify new anti-viral drugs that might be used to treat COVID-19 patients [1–5]. Remdesivir has emerged as a lead candidate with clear anti-viral activity in vitro [6] and non-human primates [7], with results in human trials suggesting benefit, although mortality remained high [8, 9]. The quest for new anti-viral drugs for SARS-CoV-2 (as for other viruses) usually begins with in vitro screening to identify potential candidates [10–12]. Initial screening usually involves assessing whether drugs can inhibit virus replication in a permissive cell line, with Vero E6 cells widely used for SARS-CoV-2. Such in vitro screening approaches often identify drugs that work well in vitro*,* but ultimately fail to have anti-viral activity in vivo. For example, chloroquine/hydroxychloroquine inhibits SARS-CoV-2 replication in vitro [6, 13, 14], but the drug ultimately emerged to have no utility in COVID-19 patients [15–17]. Chloroquine/hydroxychloroquine was similarly shown to have in vitro antiviral activity, but no anti-viral activity in humans for a number of viruses including Epstein Barr virus (infectious mononucleosis) [18], dengue [19]*,* HIV [20], chikungunya [21], Ebola [22] and influenza [23].

Although there are multiple reasons why in vitro anti-viral activity often does not translate into in vivo efficacy, one reason for false positives from in vitro screening assays is the misapplication of the therapeutic index concept as it applies to tissue culture-based anti-viral drug discovery, where this index is generally referred to as the selectivity index. The concentration of a drug that inhibits virus replication is often compared to the concentration that kills the cells (cytotoxicity). The MTS assay is also often used as a cytotoxicity or viability assay, although it actually measures mitochondrial activity. Differences in conclusions from MTS and other cytotoxicity assays are common [24], leading some to suggest complex combined cytotoxicity assays [25], which are not readily compatible with rapid screening under BSL3 containment conditions [26]. Viral replication would clearly be inhibited in cells that are not viable; however, what is perhaps under-appreciated is that viable, but stressed or otherwise slightly poisoned or compromised cells, are also likely to have a reduced capacity to replicate virus. Cellular stress responses can take multiple forms, but a key outcome of most stress responses is inhibition of translation [27–31]. Translational inhibition is also a key anti-viral response, which is able to inhibit replication of many viruses [27, 30, 32] including coronaviruses [33]. A drug that has no specific anti-viral activity, but induces cellular stress, may therefore inhibit virus replication non-specifically and generate a potential false positive in screening assays. We coin the term “cytomorbidity” to describe this phenomenon and describe herein a simple growth assay that can be used to distinguish cytomorbidity from cytotoxicity, and argue that both cytomorbidity and cytotoxicity controls are needed to increase the reliability and stringency of in vitro drug screening assays.

A key outcome of stress responses is usually to slow cell growth, allowing the cell to either recover, or if stress and/or damage is excessive, to induce cell death [34–36]. Cells that are slightly poisoned or otherwise compromised (without induction of stress responses) would likely also show reduced growth rates. Cell growth of Vero E6 cells can be very simply measured by seeding 400 cells per well in 96 well flat bottom plates and culturing with a range of drug concentrations for 4 days followed by crystal violet staining. Vero E6 cells (C1008, ECACC, Wiltshire, England; Sigma Aldrich, St. Louis, MO, USA) were plated at 4 × 10^2^ (cytomorbidity assay) or 10^4^ (anti-viral screening, cytotoxicity assay and MTS assay) cells per well in a 96 well plate in 100 µl medium and cultured overnight at 37 °C and 5% CO_2_. The drug (at 4 times the indicated final concentration) was diluted in twofold serial dilutions in RPMI 1640 supplemented with 2% FCS in a 96 well round bottom plate, and 50 µl was then transferred to cells using a multichannel pipette. For anti-viral screening assay, SARS-CoV-2 (hCoV-19/Australia/QLD02/2020 [37], kindly provided by Queensland Health Forensic and Scientific Services, Queensland Department of Health, Brisbane, Australia) was diluted in RPMI 1640 supplemented with 2% FCS to a final concentration of 2 × 10^3^ CCID_50_/ml and 50 µl was added per well using a multichannel pipette for a final MOI ~ 0.01. For cytomorbidity or cytotoxicity assay, 50 µl RPMI 1640 supplemented with 2% FCS (instead of virus) was then added per well to give a final volume of 200 µl at the desired drug concentration. The plates were cultured for 4 days at 37 °C and 5% CO_2_. To inactivate virus and stain the cells, 50 µl of formaldehyde (15% w/v) and crystal violet (0.1% w/v) (Sigma-Aldrich) was added per well to the 200 µl of medium already present in each well. After washing and drying, stain was dissolved in 100% methanol and the OD was read at 595 nm. The percentage of protein staining relative to a no-drug control was then calculated. The MTS assay was performed in duplicate where indicated using CellTiter 96 AQueous One Solution Cell Proliferation Assay (MTS) (Promega) as per manufacturer’s instructions.

**Fig. 1:**
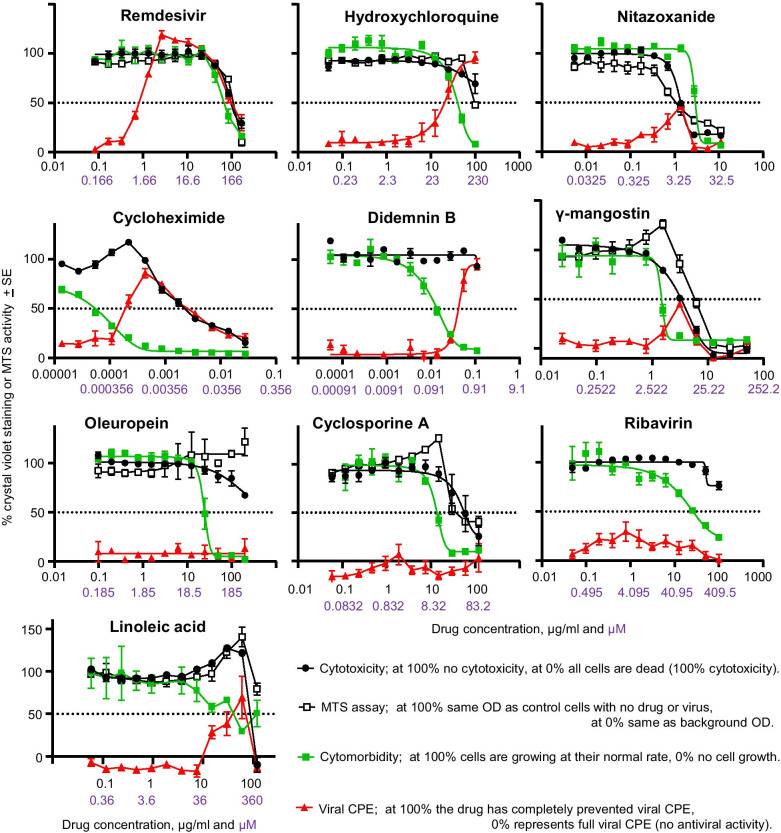
Drug cytotoxicity, cytomorbidity and inhibition of SARS-CoV-2-induced CPE. The indicated drugs at the indicated concentrations (x axis numbers µg/ml in black, µM in purple) were cultured with Vero E6 cells (i) without virus and 10^4^ cells per well to measure cytotoxicity or MTS activity (black circles and white squares), (ii) without virus and 400 cells per well to measure cytomorbidity (green squares) or (iii) with virus and 10^4^ cells per well to measure viral CPE (red triangles). Crystal violet staining was dissolved in 100% methanol and read at OD_595_. The mean percentage crystal violet staining or MTS activity (OD_490_) relative to no drug controls are shown. Error bars represent standard error of the mean (SEM) for 3–6 replicates, with each experiment undertaken independently in triplicate 1–2 times

Perhaps not surprisingly the drug concentrations that caused inhibition of cell growth were usually lower than the drug concentrations that caused cytotoxicity (Fig. 1, compare black circles with green squares). For some drugs the concentration differences for these two activities were ≥ tenfold (Fig. 1, ribavirin, cycloheximide, oleuropein, didemnin B). Inhibition of cell growth is not really cytostasis, which generally means no growth, and not really cytotoxicity, which is generally viewed as cell death. The reason(s) for reduced cell growth induced by any given drug may not be clear, and may be related to stress responses or some other phenomena that compromises the cells normal metabolic activities. Hence we suggest the term “cytomorbidity” to infer a level of cytotoxicity insufficient to kill the cells or induce cytostasis, but sufficient to stress or compromise the cells, with a simple growth bioassay used to indicate cytomorbidity. The cytomorbidity assay proposed herein, although considerably simpler, is not dissimilar in principle to a previously published cell proliferation assay used as a control for drug screening [38].

**Fig. 2:**
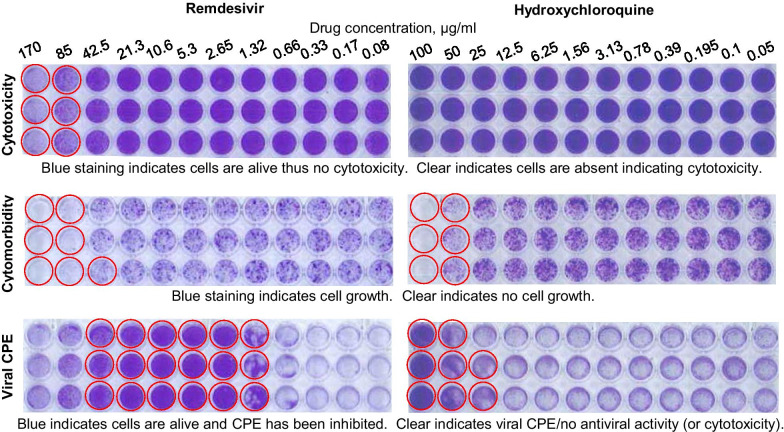
Crystal violet staining for remdesivir and hydroxychloroquine. Cytotoxicity assay (Vero E6 seeded at 10^4^ cells/well with no virus). Cytomorbidity assay (Vero E6 seeded at 400 cells/well with no virus). Viral CPE (Vero E6 seeded at 10^4^ cells/well with virus MOI ≈ 0.01). After 4 days in culture 96 well plates were fixed and stained with paraformaldehyde and crystal violet, respectively. Plates were washed in water, dried and scanned, and for the data in Fig. 1, the dye was dissolved in methanol and read at OD_595_ nm. For the Cytotoxicity assay wells encircled in red show overt cytotoxicity. For the Cytomorbidity assay wells encircled in red show overt cell growth reduction. For viral CPE assay, wells encircled in red show inhibition of CPE indicating potential antiviral activity

A simple rapid bioassay for screening drugs for potential anti-viral activity against SARS-CoV-2 is to determine whether the drug can inhibit virus-induced cytopathic effects (CPE) in Vero E6 cells. Remdesivir is known to inhibit SARS-CoV-2 replication [6] and is used herein to illustrate the behavior of an effective drug in this bioassay. Remdesivir was able to inhibit virus-induced CPE by 50% at ≈ 1 µg/ml and the drug caused 50% cytotoxicity at ≈ 100 µg/ml, providing a selectivity index of ≈ 100. Importantly, remdesivir showed cytomorbidity at ≈ 70 µg/ml, which still leaves a selectivity index of ≈ 70 (Figs. 1, 2, Table 1, Remdesivir). Hydroxychloroquine was able to inhibit viral CPE by 50% at ≈ 20 µg/ml and showed a 50% loss of viability using the MTS assay at ≈ 100 µg/ml, suggesting a selectivity index of ≈ 5. However, cytomorbidity was clearly evident at ≈ 40 µg/ml, so the anti-viral activity occurred at similar concentrations to those that caused cytomorbidity (Fig. 1, Table 1, Hydroxychloroquine); indicating a potential false positive. The overlapping activities are clearly evident when the crystal violet stained plates are viewed (Fig. 2).

**Table 1:** The half maximal inhibitory dose (IC50), half maximal cytotoxic concentration (CC50), and half maximal cytomorbidity concentration (MC50) for each compound

Drug name	IC50 (µg/ml)	IC50 (uM)	CC50 (µg/ml)	CC50 (µM)	MC50 (µg/ml)	MC50 (µM)
Hydroxychloroquine	19	43.78	NA	NA	39.8	91.72
Nitazoxanide	NA	NA	1.36	4.43	2.82	9.18
Oleuropein	NA	NA	NA	NA	24	44.40
Gamma-mangostin	NA	NA	3.31	8.35	1.47	3.70
DidemninB	0.04	0.36	NA	NA	0.013	0.12
Cycloheximide	0.0002	0.0007	0.0022	0.0078	0.000054	0.00019
Remdesivir	0.83	1.38	96.2	159.64	67.3	111.68
Ribavirin	NA	NA	NA	NA	26.1	106.88
CyclosporineA	NA	NA	57.3	47.65	13.6	11.31
Linoleic acid	17.7	63.9	40.2	144.9	18.2	65.6

The close relationship between anti-viral activity and translation inhibition (inherent in the stress responses described above) can be seen with the use of the translation inhibitors, cycloheximide and didemnin B. These drugs provide selectivity indices of ≥ 10, when comparing viral CPE inhibition and cytotoxicity. However, concentrations that inhibited viral CPE again overlapped with those that caused cytomorbidity (Fig. 1, Cycloheximide, Didemnin B). The drug γ-mangostin would appear to have a small level of anti-viral activity with a low selectivity index, but again this activity overlapped with the cytomorbidity (Fig. 1, γ-mangostin). Linoleic acid is reported to contribute to anti-viral activity at 50 µM [39]; however, this drug shows clear cytomorbidity activity above ≈ 20 µM (Fig. 1, Linoleic acid). Thus, as for hydroxychloroquine, the assay results for these latter drugs provide no supportive data for anti-viral activity, instead they suggest these drugs inhibit viral replication non-specifically by impairing cellular activities. Nitazoxanide showed some anti-viral activity, but this coincided with cytotoxicity, providing an example of the conventional cytotoxicity control that would be used to argue that the drug has no specific anti-viral activity and has a selectivity index of 1 (Fig. 1, Nitazoxanide). Curiously, higher concentrations of nitazoxanide were needed to inhibit cell growth than were needed to induce cytotoxicity; likely an example of cell density associated toxicity.

The frequently used MTS assay, as expected, often gave results similar to those provided by the cytotoxicity assay. Importantly, the MTS assay did not provide a measure of cytomorbidity, presumably because mitochondria largely remain active even in stressed cells and/or cells in G_0_ (cytostasis). For oleuropein, cyclosporine A and γ-mangostin, cytomorbidity was associated with an increase in MTS activity (Fig. 1). The MTS bioassay may thus provide slightly misleading information in this context; i.e. increased mitochondrial activity, rather than indicating increased cell numbers, can sometimes be associated with stress or mild toxicity.

The CPE-based assay described herein has some inherent limitations. Drugs whose mechanism of action require induction of type I interferons, would be ineffective in this assay system as Vero E6 cells do not make type I interferons. The CPE-based assay also provides a low sensitivity read-out. Higher drug concentrations are likely needed to prevent virus-induced CPE (overwhelming infection resulting in cell death) than would be needed to inhibit viral replication as measured (for instance) by qRT-PCR of virus released into culture supernatants [40]. Although more sensitive anti-viral activities exist [40], the CPE-based assay represents a screening tool able rapidly and cheaply to identify promising anti-viral candidates. More sensitive assays could be also envisaged for assessing cytomorbidity, such as measuring activation of stress factors such as ATF3 [41], analyzing cell cycle perturbations by flow cytometry or cell growth kinetics using the IncuCyte live-cell analysis system. The cell line used herein, Vero E6, is a monkey kidney-derived cell line, whereas in humans ciliated airway cells and alveolar type II pneumocytes (AT-2 cells) are thought to be the primary targets for SARS-CoV-2 infection [42]. Drug metabolism and/or bioavailability in such cells may not be reflected in Vero E6 cells. However, although a number of human cell lines support SARS-CoV-2 infection, few if any exhibit the fulminant CPE seen in Vero E6 cells [43].

## Conclusions

In conclusion, in vitro screening of anti-SARS-CoV-2 drugs should include not just a cytotoxicity control, but also a cytomorbidity control in order to identify potential false positives associated with anti-viral activity arising from non-specific stress responses or other disruptions of cellular activities/functions.

## Supplementary Information

**Additional file 1.** Extended methods.

## Data Availability

All data generated or analysed during this study are included in this published article and its Additional file 1.

## Abbreviations

CPE: Cytopathic effects
HIV: Human immunodeficiency virus
MTS: 3-(4,5-Dimethylthiazol-2-yl)-5-(3-carboxymethoxyphenyl)-2-(4-sulfophenyl)-2*H-*tetrazolium
BSL3: Biosafety level 3
RPMI: Roswell Park Memorial Institute medium
FCS: Fetal calf serum
CCID50: Cell culture infectious dose 50%
OD: Optical absorbance
qRT-PCR: Real-time quantitative reverse-transcriptase polymerase chain reaction
