# Supplementary material for: Simple rapid in vitro screening method for SARS-CoV-2 anti-virals that identifies potential cytomorbidity-associated false positives
Source: Virol J. 2021 Jun 9;18:123. doi: 10.1186/s12985-021-01587-z (PMC8188739; doi:10.1186/s12985-021-01587-z)
Supplement: Supplementary file 1 — Additional file 1. Extended methods. [file 12985_2021_1587_MOESM1_ESM.docx]

**Extended Methods**

**PC3/BSL3 facilities.** All infectious SARS-CoV-2 work was conducted in a dedicated suite within the PC3/BSL3 facility at the QIMR Berghofer MRI (Australian Department of Agriculture, Water and the Environment certification Q2326 and Office of the Gene Technology Regulator certification 3445). Work was approved by the QIMR Berghofer MRI Biosafety Committee (P3600).

**Cells and SARS-CoV-2 virus.** Vero E6 cells (C1008, ECACC, Wiltshire, England; Sigma Aldrich, St. Louis, MO, USA) were cultured in medium comprising RPMI1640 (Gibco) supplemented with 10% fetal calf serum (FCS), penicillin (100 IU/ml)/streptomycin (100 μg/ml) (Gibo/Life Technologies) and L-glutamine (2 mM) (Life Technologies). Cells were routinely checked for mycoplasma (MycoAlert Mycoplasma Detection Kit MycoAlert, Lonza) and FCS was assayed for endotoxin contamination before purchase {Johnson, 2005 #33}. The SARS-CoV-2 virus was kindly provided by Queensland Health Forensic & Scientific Services, Queensland Department of Health, Brisbane, Australia. The virus (hCoV-19/Australia/QLD02/2020) was isolated from a patient and sequence deposited at GISAID (<https://www.gisaid.org/>; after registration and login, sequence can be downloaded from <https://www.epicov.org/epi3/frontend#1707af>). Virus stock was generated by infection of Vero E6 cells at multiplicity of infection (MOI)≈0.01, with supernatant collected after 3 days, cell debris removed by centrifugation at 3000 x g for 15 min at 4°C, and virus aliquoted and stored at -80 °C. Virus titers were determined using standard CCID_50_ assays (see below). The virus was determined to be mycoplasma free using co-culture with a non-permissive cell line (i.e. HeLa) and Hoechst staining as described {La Linn, 1995 #34}.

**Virus titration by CCID_50_ assay.** Virus was titrated using a standard Cell Culture Infectivity Dose 50 (CCID_50_) assay. Vero E6 cells were plated into 96 well polystyrene flat bottom plate (Sigma Aldrich) at 2x10^4^ cells per well in 100 µl of medium (see above). The following day 100 µl of virus was added in 10 fold serial dilutions in RPMI 1640 supplemented with 2% FCS, and the plates cultured for 4 days at 37°C and 5% CO_2_. The presence of cytopathic effect (CPE) was determined using an inverted light microscope in a biosafety cabinet attached to a screen, and the virus titer determined by the method of Spearman and Karber (a convenient Excel CCID_50_ calculator is available at <https://www.klinikum.uni-heidelberg.de/zentrum-fuer-infektiologie/molecular-virology/welcome/downloads>).

**Drugs.** Didemnin B was kindly provided by the Natural Products Branch, NCI (NSC 325319, Bethesda, MD, USA). Cycloheximide, nitazoxanide, ribavirin, hydroxychloroquine sulfate, γ-mangostin and oleuropein were all purchased from Sigma Aldrich. Remdesivir was purchased from AdooQ BioScience. Cyclosporine A (Merck Millipore) was dissolved in 100% ethanol. Ribavirin and hydroxychloroquine sulfate was dissolved in Ultrapure Distilled Water (Life Technologies). All other drugs were dissolved in DMSO (Sigma Aldrich). All drugs were aliquoted and stored at -80°C. Drugs were tested in duplicate or triplicate in 1 to 3 independent experiments. For cytotoxicity, cytomorbidity and anti-viral activity the total number of replicates were, respectively: 5, 4, and 3 remdesivir; 8, 5 and 6 hydroxychloroquine, 8, 8, and 6 nitazoxanide; 3, 3 and 3 cyclohexamide, 3, 3 and 3 didemnin B; 6, 6 and 3 γ-mangostin; 5, 5 and 3 oleuropein; and 5, 2, and 3 for cyclosporine A and ribavirin.

**Crystal violet staining.** To inactivate virus and stain the cells, 50 µl of formaldehyde (15% w/v) and crystal violet (0.1% w/v) (Sigma-Aldrich) was added per well to the 200 µl of medium already present in each well. Plates were left overnight inside a closed biosafety cabinet with lids on (biosafety cabinet UV switched on for 20 mins). The plates (lids off) and inverted lids were then exposed to 7.6 kJ/m2 UV-C. The lids were replaced and the plates sprayed with 80% (v/v) ethanol; plates were labelled with Alcohol Resistant Cryogenic Permanent Markers (Science Marker). The plates were then deemed decontaminated and removed from the PC3/BSL3 facility. The plates were washed in tap water, dried overnight (photographed or scanned) and 100 µl/well of 100% methanol added to dissolve the crystal violet and the OD was read at 595 nm using a 96-well plate reader (Biotek Synergy H4).

**MTS assay.** The MTS assay was performed in duplicate where indicated using CellTiter 96 AQueous One Solution Cell Proliferation Assay (MTS) (Promega) as per manufacturer’s instructions. Briefly, 40 µl of CellTiter 96 AQueous One Solution Reagent was added per well. Cells were incubated at 37 °C and 5% CO_2_ until color change was visible (approximately 1 hr). 100 µl of cell culture supernatant was then transferred to a new 96 well plate and absorbance at 490 nm was read using a 96-well plate reader (Biotek Synergy H4).

**SARS-CoV-2 antiviral screening assay, drug cytomorbidity and cytotoxicity assay**

1. Vero E6 cells were plated at 4×10^2^ (cytomorbidity assay) or 10^4^ (cytotoxicity assay) cells per well in a 96 well plate in 100 µl medium and cultured overnight at 37 °C and 5% CO_2_.
2. The drug (at 4 times the indicated final concentration) was diluted in 2 fold serial dilutions in RPMI 1640 supplemented with 2% FCS in a 96 well round bottom plate, and 50 µl was then transferred to cells using a multichannel pipette.
3. For anti-viral screening assay, SARS-CoV-2 virus was diluted in RPMI 1640 supplemented with 2% FCS to a final concentration of 2×10^3^ CCID_50_/ml and 50 µl was added per well using a multichannel pipette for a final MOI~0.01. For cytomorbidity or cytotoxicity assay, 50 µl RPMI 1640 supplemented with 2% FCS (instead of virus) was then added per well to give a final volume of 200 µl at the desired drug concentration.
4. The plates were cultured for 4 days at 37 °C and 5% CO_2_.
5. The MTS assay was performed as described in the ‘MTS assay’ section, followed by crystal violet staining as described in the ‘crystal violet staining’ section.

**Calculations**

1. Background; the mean background represented the mean of OD values from 6 wells with cells (no drug) infected with virus (100% CPE or 0% staining).

2. Control: for antiviral and cytotoxicity assays, the mean control OD represented the mean of OD values from 6 wells with cells (no drug) and no virus (0% CPE or 100% staining).

3. A percentage was calculated for each well using (Sample OD – Background OD) / (Control OD – Background OD) x 100, and a mean and standard error obtained from the replicates.

For cytomorbidity and MTS assays the background was the mean of OD values from wells with medium but no cells (no drug, no virus) (0% staining/MTS activity).
